# Retraction: Topical application of daphnetin hydrogel for traumatic brain injury

**DOI:** 10.3389/fnins.2025.1751561

**Published:** 2025-11-28

**Authors:** Frontiers Editorial Office

The journal retracts the August 7 2024 article cited above.

Following publication, concerns were raised regarding the integrity of the images in the published figures. The authors failed to provide a satisfactory explanation during the investigation, which was conducted in accordance with Frontiers' policies.

This retraction was approved by the Chief Executive Editor of Frontiers. The authors received a communication regarding the retraction and had a chance to respond. This communication has been recorded by the publisher.

Frontiers would like to thank Dr. Sholto David for bringing the published manuscript to our attention.

